# Simulation of a Miniature Linear Ion Trap with Half-Round Rod Electrodes

**DOI:** 10.3390/mi13101572

**Published:** 2022-09-22

**Authors:** Xichi Lu, John T. W. Yeow, Gongyu Jiang, Yu Xiao, Rujiao Yao, Qi Zhang, Jiacheng Song, Jinyuan Yao

**Affiliations:** 1 National Key Laboratory of Science and Technology on Micro/Nano Fabrication, Shanghai Jiao Tong University, Shanghai 200240, China; 2 Department of Micro/Nano Electronics, School of Electronic Information and Electrical Engineering, Shanghai Jiao Tong University, Shanghai 200240, China; 3 Systems Design Engineering, University of Waterloo, Waterloo, ON N2L 3G1, Canada; 4 Institute of Spacecraft Equipment, Shanghai 200240, China

**Keywords:** linear ion trap, theoretical simulation, mass resolution, round rod electrodes, MEMS

## Abstract

The miniaturization of ion trap mass analyzers is an important direction in the development of mass spectrometers. In this work, we proposed two models of miniaturized HreLIT with a field radius of about 2 mm based on the existing research on conventional HreLIT and other ion traps, one with ions ejection slits on one pair of electrodes only (2-slit model) and the other with the same slits on all electrodes (4-slit model). The relationship of mass resolution with *r/r_x_* and the “stretch” distance of electrodes in the ejection direction is investigated by theoretical simulations. Trends of electric fields inside the ion traps were discussed as well. The comparable maximum resolution is observed at *r/r_x_* = 2/1.4 in both models, but stretching simulations revealed that the peak resolution of the 2-slit model was higher than that of the other model by about 8%. The highest value of 517 was obtained when stretching 1.1 mm. Furthermore, the resolution of ions with *m*/*z* = 119 could exceed 1000 when the scan rate was reduced to 800 Th/s. The mass spectrometry capability of miniature HreLIT has been confirmed theoretically, and it laid the foundation for the subsequent fabrication with MEMS technology.

## 1. Introduction

Mass spectrometry (MS) is an essential analytical technique for measuring the *m*/*z* of ions. Recent developments in mars and lunar exploration [1,2] and public health emergencies [3] have heightened the need for portable mass spectrometers capable of rapid on-site detecting, and the miniaturization of mass analyzers is the key to reducing the size of MS devices. Ion traps consist of simple structures, possessing the capability to multi-stage tandem mass analysis with only one single device, and are superior in miniaturization compared with other mass analyzers. In addition, the low requirements for the supporting experimental conditions are of benefit to satisfying the needs of diverse real-time analysis.

The three-dimensional (3D) ion trap reported by Paul [4] and the two-dimensional linear ion trap (LIT) proposed by Schwartz [5] laid the foundation for modern research of ion trap mass analyzers. The latter provided higher ion capacity and trapping efficiency. Both ion traps were built with hyperbolic-shaped electrodes that require extremely high machining and assembly accuracy, and the performance is sensitive to machining errors. Therefore, a growing number of ion traps with simplified electrodes based on the two models were reported to reduce the difficulties of preparation with acceptable performance loss. For example, a cylindrical ion trap [6,7] (CIT) was proposed to simplify the electrodes of a 3D ion trap; a rectilinear ion trap [8] (RIT) with planar-shaped electrodes combined some advantages of LIT and CIT; a triangular-electrode linear ion trap [9] (TeLIT) was built by modifying RIT, etc. Offering a resolution higher than 4000, the LIT-based half-round rod electrodes linear ion trap (HreLIT) reported by Li et al. [10] is conducive to fabricating compact analyzers. Various novel ion traps with electrodes that are easy to prepare could have significant applications for miniaturization.

The common ion traps with simplified electrodes were reported with field radii ranging from 4 mm to 6 mm and have been applied in benchtop or over-the-shoulder mass spectrometers. Despite the certain size reduction, there has been an increasing interest in high-performance palm portable mass spectrometers with miniature mass analyzers because of nowadays timely detection requirements under complicated conditions. For example, a palm portable mass spectrometer (PPMS) reported by Sam Yang Chemical Company [11] has a size of 82 × 77 × 245 mm; the MX908 developed by 908 Devices [12] is a little bigger but just 298 × 216 × 122 mm in size, both instruments are capable of detecting environmental components with single-handed operation. In order to miniaturize ion traps, micro electro mechanical system (MEMS) technology, which could break the bottleneck of conventional processing methods, has been exerting a tremendous fascination on researchers. In this case, a Halo ion trap [13] with a field radius of only 0.5 mm was fabricated with MEMS technology, but the low resolution was confirmed; Yu et al. [14] reported an RIT with a field radius of 1.5 mm, being built by stereo lithography apparatus (SLA) and offering a slightly higher resolution of 100; Huang and co-worker [15] optimized a MEMS-based RIT by working on the relationships of ion trapping efficiency with length–width ratio and radio frequency (RF) voltage; Szyszka et al. [16] combined the MEMS with 3D printing technology successfully to fabricate a miniature quadrupole mass analyzer, but this structure has lower resolution and ion capacity compared with ion traps. The miniature RIT array proposed in our laboratory [17] was prepared by MEMS technology, and the resolution could exceed 700. However, there remains much room for improvement compared with ion traps in conventional size. Definitely, MEMS technology plays a prominent role in the miniaturization of mass analyzers, and there exists an obvious conflict between mini size and high performance. The exploration of more balanced miniature ion traps with different structures is indispensable.

LIT, CIT, and RIT are widely applied in miniaturized ion traps. Although CIT facilitates miniaturization, the physical limits of 3D ion traps determine the low resolution and charge capacity. For LIT and RIT, on the one hand, the closer the shape of electrodes is to the hyperboloid, the more ideal the quadrupole field will be. On the other hand, the mass resolution could be improved significantly by optimizing the geometric parameters in a fixed shape of electrodes [5,18]. The excessive high-order fields introduced by the oversimplified planar-shaped electrodes of the RIT are more pronounced at micro size, and in consequence, the resolution would be limited to an unremarkable range even with sufficient optimization [17]. Meanwhile, the promising HreLIT with a field radius of about 5 mm provides excellent MS capability, and the performance could be further improved after optimization [19,20]. Its performance at micro size is worth investigating accordingly.

In the present work, the miniature HreLIT with a field radius of about 2 mm was modeled and optimized, referring to the methods applied by conventional ion traps. How the slits on electrodes, field radius, and “stretch” distance of electrodes in ejection direction affect, MS capability was investigated by computer simulations. The objective was to explore the feasibility of miniaturizing HreLIT and lay the foundation for future fabrication based on the non-silicon MEMS technology in our laboratory.

## 2. Simulation Methods

### 2.1. Calculation of Electric Fields

The motion of ions in HreLIT would be determined directly by the internal electric fields. Its two-dimensional electric potential *φ(x*, *y)* satisfies the Laplace equation:

(1) $$\nabla^{2}\varphi (x,\;y)=0,$$

and there is no analytical solution, and *φ(x*, *y)* could be described by the real part of the accumulation of fields [18]:

(2)  φ(x, y)=VRFRe[∑n=0∞An(x+iyr0)n], n∈ N,

where *r*_0_ is the field radius, *V_RF_* means the amplitude of RF voltage, *A_n_* represents the amplitude of 2n-order fields, and *i* is the imaginary unit.

SIMION (Adaptas Scientific Instrument Services (Adaptas SIS), Palmer, MA, USA) could solve the approximate potential at each lattice point inside the model by the finite difference method (FDM) and generate potential array (PA) files. Then the fast Fourier transform (FFT) in PAN33 [21] provides an efficient approach to calculating the high-order fields according to it.

### 2.2. Modeling and Simulation

Optimizing the fields remains a key process in the design of ion traps since appropriate high-order fields could be mutually compensated [18] and improve the MS capability. For this purpose, adjusting the ratio of electrode size to field radius (*r/r*_0_) and “stretching” electrodes in the ejection direction were reported to be effective. Douglas and co-workers [18] indicated that the *r/r*_0_ of LIT with round rod electrodes significantly affected the performance. Schwartz and co-workers [5] “stretched” the electrodes in the ejection direction by 0.75 mm away from the geometric center to compensate for the fields, and this approach was referred to in our research.

**Figure 1:**
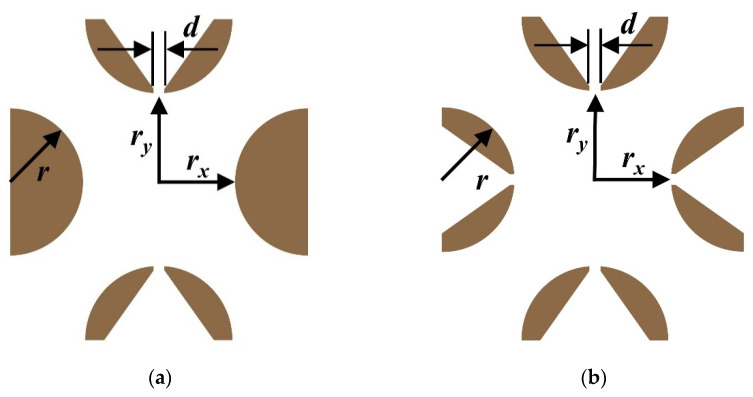
Cross-section of miniature HreLIT. (**a**) 2-slit model; (**b**) 4-slit model.

Therefore, with a fixed electrode radius, the effect of varying the field radius and “stretching” the electrodes in the ejection direction on the resolution was investigated by calculating fields and simulating the motion of ions. In view of the harmful high-order fields introduced by slits on electrodes [10,22], two HreLIT models have been developed for discussion according to the TeLIT with four slits [9], one with ions ejection slits on one pair of electrodes only (2-slit model, Figure 1a) and the other with the same slits on all electrodes (4-slit model, Figure 1b). The width of slits (*d*) was 300 μm. The radius of all electrodes represented by *r* remained 2 mm. The shortest distance from electrodes to the geometric center (field radius) was *r_x,_* and that from the electrodes in the ejection direction was *r_y_*.

Describing the models with Geometry Files, the potential inside ion traps was solved by SIMION 8.1, and the following PA files were generated. Based on this, the fields and motion of ions have been calculated with PAN33 and AXSIM (PAN33 version 3.3.5 and AXSIM version 5.9.0 were developed by Shimadzu Research Laboratory (Europe) Ltd, Manchester, United Kingdom.), respectively.

When simulated with a hard sphere collision model, ions (*m*/*z* = 117, 119, and 121, each with the number of 100) with no initial velocity were placed close to the geometric center randomly. The helium (300 K, 0.13 Pa) was used as a buffer gas. The AC signal was applied to electrodes in the y direction, and the RF signal, which confined ions and altered their vibrational frequency, was applied between the electrodes in the x and y directions. The frequency of AC was one-third of that of RF, and the scan rate was 2250 Th/s. As the amplitude of the RF signal increased, consequently, ions were excited and ejected from the slits in sequence because of matched frequencies of vibration and the AC signal. AXSIM analyzed this process and recorded the time and positions of ejected ions. These data were read by IC5Filter, and mass spectrums, which were used to estimate MS capability, were output immediately. In this way, the optimal resolution corresponding to *r/r_x_* was found by continuously changing *r_x_*. Then the “stretch” simulations of *r_y_* at this *r/r_x_* were performed likewise, and finally, the relationships between mass resolution and the parameters above were obtained.

## 3. Results and Discussion

### 3.1. The Impact of r/r_x_

**Figure 2:**
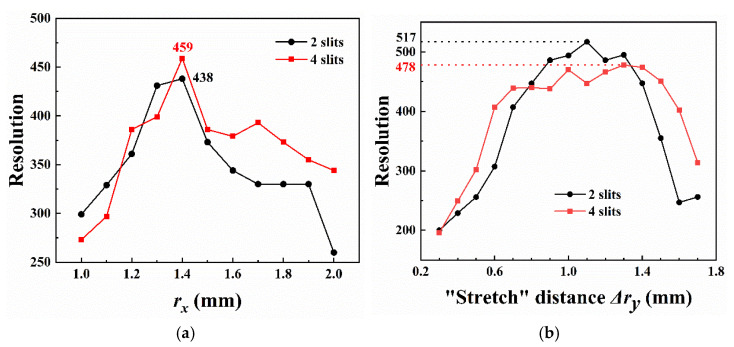
(**a**) Trends of mass resolution with *r_x_* at Δ*r_y_* = 0.75 mm; (**b**) trends of mass resolution with Δ*r_y_* at *r/r_x_* = 2/1.4. Appropriate field radius *r_x_* and “stretch” distance Δ*r_y_* led to better resolutions.

**Figure 3:**
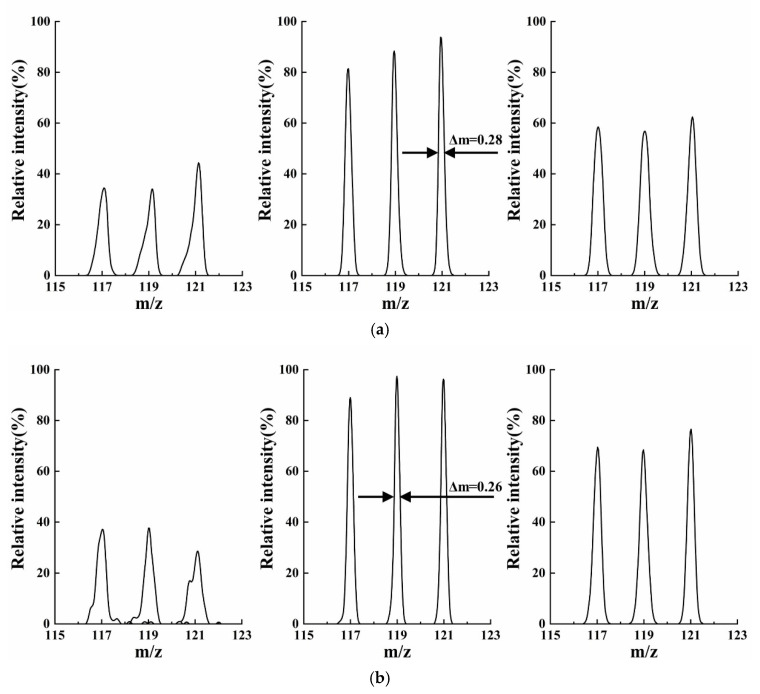
Mass spectrum when *r/r_x_* = 2/1, 2/1.4, and 2/2 with Δ*r_y_* kept 0.75 mm. (**a**) 2-slit model; (**b**) 4-slit model. Not only poor resolutions but also lower intensity and even distortion were observed when field radius *r_x_* was too big or too small.

The “stretch” distance Δ*r_y_* kept 0.75 mm (*r_y_* = *r_x_* + 0.75) and *r_x_* was extracted at intervals of 0.1 mm within 1.0~2.0 mm. The mass resolution trends are shown in Figure 2a according to AXSIM calculating the motion of ions at a scan rate of 2250 Th/s. A comparison of the mass spectrum at *r/r_x_* = 2/1, 2/1.4, and 2/2 (Figure 3) showed the low resolution and intensity when the *r_x_* was extracted at the interval endpoints. Even a significant distortion could be observed in the 4-slit model. For both cases, the comparable maximum resolution with high intensity, 438 and 459, respectively, were measured at *r/r_x_* = 2/1.4.

**Figure 4:**
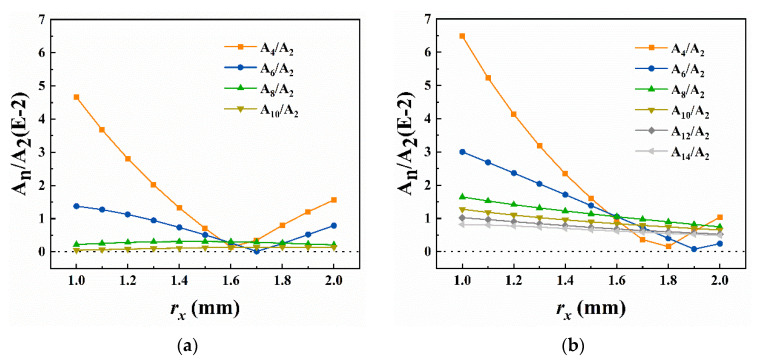
Variation of the high-order fields with *r_x_* at Δ*r_y_* = 0.75 mm. (**a**) 2-slit model; (**b**) 4-slit model. More slits on electrodes introduced higher-order fields and increased the overall contents and higher rate of changes.

Figure 4 shows the proportions of high-order fields. The A_4_/A_2_ and A_6_/A_2_ of both models changed remarkably with increasing *r_x_*. Different from the V-shape trend, the higher-order fields provided smooth curves at lower levels. Obviously, there existed more high-order fields in the 4-slit model since the extra slits led to more severe distortions. Within 1.0~1.4 mm, the resolution increased due to the rapid decrease in A_4_/A_2_ and A_6_/A_2_, but neither model showed the best resolution when A_4_/A_2_ and A_6_/A_2_ tended to 0. It probably owed to the non-negligible higher-order fields (such as A_8_/A_2_ and A_10_/A_2_), which became the dominant factor affecting the MS capability at this *r_x_*.

### 3.2. The Impact of “Stretch” Distance

The “stretch” simulations of the y electrodes were performed with *r_x_* being fixed at 1.4 mm according to the previous results. To avoid serious distortions in the mass spectrum, the “stretch” distance Δ*r_y_* is extracted at intervals of 0.1 mm within 0.3~1.7 mm (the correspondences between Δ*r_y_* and *r_y_* are shown in Table 1). AXSIM calculated the motion of ions at 2250 Th/s, and resolution trends were graphed, as shown in Figure 2b. Both models showed an overall trend of increasing, stabilizing, and decreasing with the increase in Δ*r_y_*. For the 2-slit model, the resolution exceeded 400 when Δ*r_y_* was between 0.7 mm and 1.4 mm, with the highest value of 517 observed at Δ*r_y_* = 1.1 mm. Differently, the peak resolution of the 4-slit model was obtained at Δ*r_y_* = 1.3 mm, which corresponded to the value of 478, but a mass resolution higher than 400 could be measured in a larger range (0.6~1.6 mm) of Δ*r_y_*.

**Figure 5:**
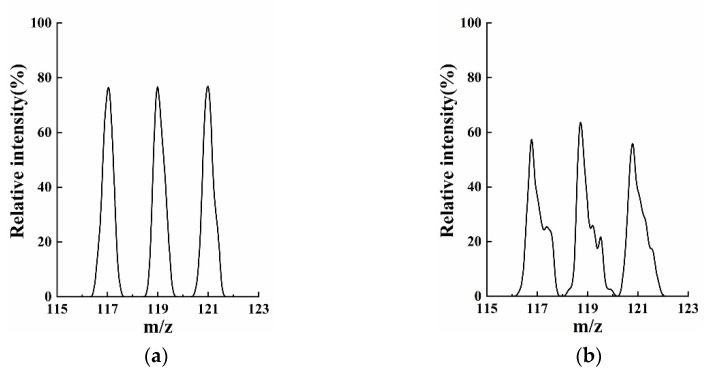
Mass spectrum of 2-slit model (*r_x_* = 1.4 mm) when (**a**) Δ*r_y_* = 1.6 mm; (**b**) Δ*r_y_* = 1.7 mm. Lower full width at half maximum (FWHM) with severe distortion limited the MS capability.

It should be noted that the 2-slit model at Δ*r_y_* = 1.7 mm did not perform better than at Δ*r_y_* = 1.6 mm, as shown in Figure 2, because the mass spectrum manifested a severe distortion at Δ*r_y_* = 1.7 mm, a phenomenon that did not occur at Δ*r_y_* = 1.6 mm (Figure 5).

**Figure 6:**
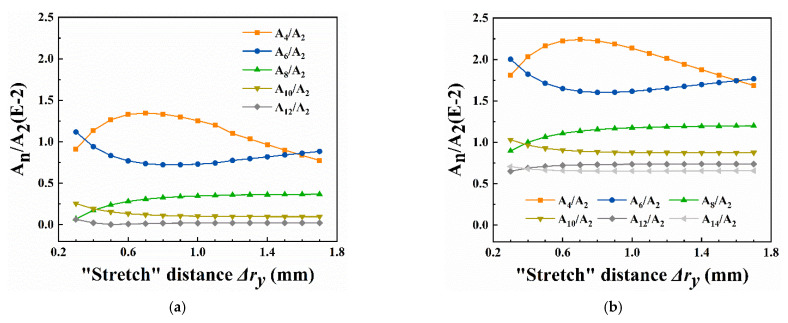
Variation of the high-order fields with Δ*r_y_* at *r_x_* = 1.4 mm. (**a**) 2-slit model; (**b**) 4-slit model. More slits on electrodes introduced higher-order fields and increased the overall contents.

The proportions of high-order fields are shown in Figure 6. In the 2-slit model, the A_4_/A_2_ and A_6_/A_2_ dominating MS capability kept at a high level and changed apparently during the electrodes “stretching” process. The A_8_/A_2_ and higher-order fields were relatively low and varied slowly, so their impact on resolution, in contrast, was negligible. When Δ*r_y_* ranged within 0.9~1.3 mm, where A_4_/A_2_ decreased rapidly with A_6_/A_2_ increasing slightly, the 2-slit model offered almost the same high resolution. It is probably because the A_4_/A_2_ and A_6_/A_2_ provided commendable compensation for high-order fields in this case, with 1.2% A_4_/A_2_ and 0.74% A_6_/A_2_ corresponding to the peak resolution of 517. As for the 4-slit model, the fields higher than A_6_/A_2_ remained steady as well. The high resolution could be observed with Δ*r_y_* ranging from 0.7 mm to 1.5 mm, a longer closed interval than in the 2-slit model. The appropriate A_4_/A_2_ and A_6_/A_2_ could likewise explain these similar trends in Figure 2b.

Comparing the proportion curves of high-order fields in detail, it could be found that more harmful fields were introduced in the model with four slits on electrodes. For example, its peak of A_4_/A_2_ was 0.9% higher than that in the 2-slit model and reached 2.24%; A_10_/A_2_, A_12_/A_2,_ and A_14_/A_2_ increased significantly, even doubling or tripling. More unexpected high-order fields led to a slightly lower peak resolution of 478 for the 4-slit model than 517 for the 2-slit model in “stretch” simulations.

### 3.3. Further Investigation of MS Capability

**Figure 7:**
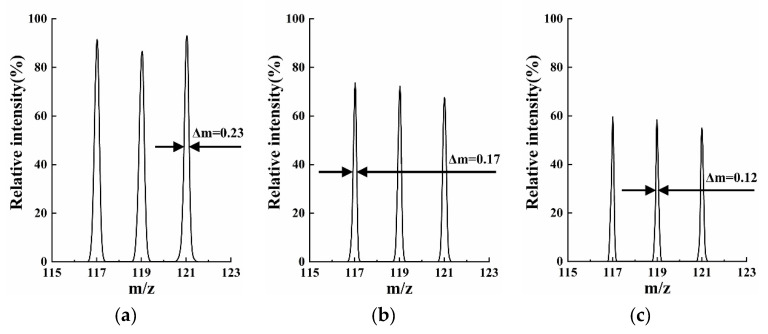
Mass spectrum and FWHM of the 2-slit model (*r_x_* = 1.4 mm, *r_y_* = 2.5 mm) at different scan rate: (**a**) 2250 Th/s; (**b**) 1250 Th/s; (**c**) 800 Th/s. The corresponding resolutions were 517, 670, and 1017.

The scan rate [23] plays an important role in MS capability, and its impact should be paid attention to. In practice, researchers would change the scan rate to obtain high resolution or intensity according to requirements. A high scan rate provides higher intensity sacrificing mass resolution, while a lower scan rate is the opposite. However, analyzing with an extremely low scan rate would lead to unacceptable intensity and serious distortion in the mass spectrum instead of a remarkable resolution. All the previous simulations were performed at the scan rate of 2250 Th/s. Based on the 2-slit model with the best performance in the simulations above (*r_x_* = 1.4 mm, *r_y_* = 2.5 mm), we investigated the MS capability at a lower scan rate while other parameters were kept unchanged. As shown in Figure 7, at 1250 Th/s and 800 Th/s, the full width at half maximum (FWHM) decreased to 0.17 and 0.12, respectively, and the corresponding mass resolutions were 670 and 1017.

In a similar size, the previously proposed miniature four-channel RIT [17] (*r_x_* = 1.4 mm, *r_y_* = 1.61 mm of a single channel) in our laboratory optimized the proportions of high-order fields and improved the analysis performance compared with the single-channel one, but at the cost of almost quadrupled volume and higher amplitude or frequency of the RF signal. The mass resolution of miniature HreLIT in this work with a tiny size between the single-channel and four-channel RIT was the most outstanding in these three ion traps. The merits of cambered electrodes were proved again compared with planar-shaped electrodes in the miniaturization of ion traps.

## 4. Conclusions

In order to verify the feasibility of developing miniature HreLIT, two models with two slits and four slits on electrodes, respectively, were proposed and investigated by theoretical simulations. The results indicated that at 2250 Th/s, both models performed comparably and excellently when *r/r_x_* = 2/1.4. Furthermore, the “stretch” simulations on this basis manifested that the 2-slit model possessed about 8% higher peak resolution, but the 4-slit model performed well in 0.6~1.6 mm of Δ*r_y_*, a wider range compared with 0.7~1.4 mm. The highest resolution of 517 was obtained by the 2-slit model with Δ*r_y_* = 1.1 mm (*r_y_* = 2.5 mm). This value could be significantly improved with a reduction in scan rate at the acceptable cost of intensity. Consequently, the resolution of 1017, 44% higher than that of the previous miniature four-channel RIT, was observed at a scan rate of 800 Th/s. In future research, MEMS technology will be applied to fabricate the miniature HreLIT, and its MS capability will be characterized experimentally.

## Figures and Tables

**Table 1:** The partial correspondences between Δ*r_y_* and radius.

Radius (mm)	Δ*r_y_* (mm)							
	0.3	0.5	0.7	0.9	1.1	1.3	1.5	1.7
*r_x_*	1.4	1.4	1.4	1.4	1.4	1.4	1.4	1.4
*r_y_*	1.7	1.9	2.1	2.3	2.5	2.7	2.9	3.1

## Data Availability

All data for final conclusions are present in this paper. Additional related information may be requested from the authors.
